# Impacts of Residual Stress on Micro Vibratory Platform Used for Inertial Sensor Calibration

**DOI:** 10.3390/s20143959

**Published:** 2020-07-16

**Authors:** Rui Hao, Huijun Yu, Bei Peng, Haixiang Zhan, Wu Zhou

**Affiliations:** School of Mechanical and Electrical Engineering, University of Electronic Science and Technology of China, Chengdu 611731, China; hr1351440347@std.uestc.edu.cn (R.H.); yuhjuestc@126.com (H.Y.); beipeng@uestc.edu.cn (B.P.); zhanhaixiang@163.com (H.Z.)

**Keywords:** micro vibratory platform, inertial sensor calibration, residual stress, PZT hysteresis

## Abstract

A micro vibratory platform driven by converse piezoelectric effects is a promising in-situ recalibration platform to eliminate the influence of bias and scale factor drift caused by long-term storage of micro-electro–mechanical system (MEMS) inertial sensors. The calibration accuracy is critically determined by the stable and repeatable vibration of platform, and it is unavoidably impacted by the residual stress of micro structures and lead zirconate titanate (PZT) hysteresis. The abnormal phenomenon of the observed displacement response in experiments was investigated analytically using the stiffness model of beams and hysteresis model of piezoelectric material. Rather than the hysteresis, the initial deflection formed by the residual stress of the beam was identified as the main cause of the response error around the zero position. This conclusion provides guidelines to improve the performance and control of micro vibratory platforms.

## 1. Introduction

Micro-electro–mechanical system (MEMS) inertial sensors are widely used in the aerospace field [1] and in intelligent robots [2]. However, its bias and scale factor drift caused by temperature [3] and long-term storage [4,5] prevent its applications in highly precise fields [6]. Therefore, an in-situ and movable recalibration platform or method is much needed to suppress the drift without relying on fixed equipment [7] and strict environmental requirements [8]. To meet these requirements, a micro vibratory platform driven by converse piezoelectric effect was proposed to provide an inertial stimulus for both gyroscope [9] and accelerometer [10] calibration. Compared with the self-test structure that is usually used for evaluating the device performance [11], the vibratory platform can generate an accurate signal to quantitatively calibrate the bias and scale factor of sensors.

A multi-axis vibratory platform designed by a team at the University of Michigan for in situ self-calibration of general MEMS inertial sensors [12,13], which can achieve 150°/s angular rate and 0.3 g (1 g = 9.8 m/s^2^) linear acceleration. It is integrated with the commercial Invensense MPU-6500 (IMU) and is expected to provide the scale-factor calibration with estimation error <20 ppm, yet the capacitive position sensor for platform motion detection has temperature drift [14] and dielectric polarization effect [15]. After that, a team at Cornell University reported a new vibratory platform for gyroscope calibration [16], which adds an integrated optical metrology system and a CMOS (Complementary Metal Oxide Semiconductor) imager for closed loop control of the vibratory platform. The piezoelectric vibratory platform is capable of generating 0~300°/s angular rate for scale factor and bias measurement, and it has 0~90 m/s^2^ in-plane acceleration to extract the gyroscope in-plane acceleration sensitivities. The additional optical metrology system can ensure the long-term stability of the vibratory platform at 10 ppm. In the same period, a team at the China Academy of Engineering Physics also reported a micro vibratory platform with high dynamic for the in-situ calibration of inertial MEMS devices [17]; it can provide 16 g acceleration and 720°/s angular rate for 19 mg payload (a commercial 3-axis accelerometer H3LIS331DL).

However, due to the appearance of the residual stress of micro fabrication and the hysteresis effect [18,19] of piezoelectric material, the vibratory stage exhibited an initial displacement of 0~20 μm and an abnormal displacement response, which was obtained using an optical measurement system proposed in our previous work [20]. After that, the platform was optimized to gain a lower off-axis error less than 1% [21], but the improvement contributed little to reducing the error induced by residual stress and the hysteresis effect.

This study aimed to find out the underlying mechanism behind the abnormal z-axis displacement curve of the platform test. The test results are shown in Section 2 to show which error needs to be studied, and Section 3 describes how the investigation was carried out in detail. In Section 4, the influence of residual stress on the vibratory platform displacement response is discussed farther. Finally, a conclusion is included in Section 5.

## 2. Platform Test

**Figure 1:**
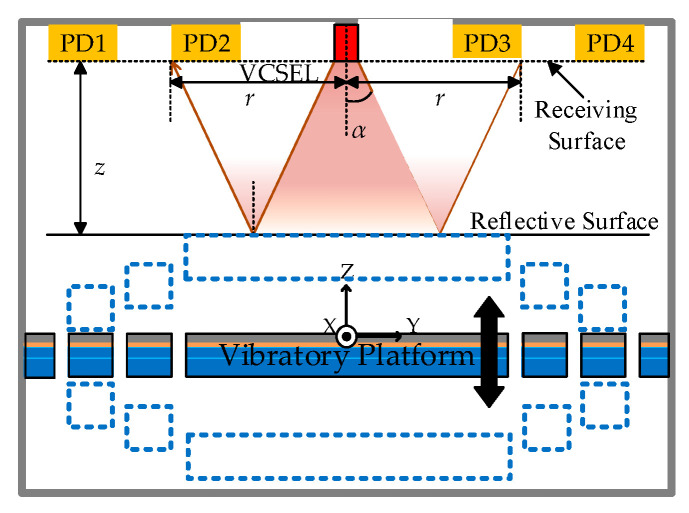
Schematic diagram of the optical measurement system.

The optical test method [20] and fabrication [21] of a vibratory platform were reported in recent work. The measurement principle is as follows. It consists of a central vertical cavity surface emitting laser (VCSEL) and several photodiodes (PDs); when the vibratory platform vibrates along the *z*-axis, the relative movement between the vibratory platform surface and the light receiving surface of optical measurement system affects the intensity of reflected light from VCSEL. Then, the reflected lights can be extracted by the PDs array to achieve accurate motion perception and estimation (Figure 1).

**Figure 2:**
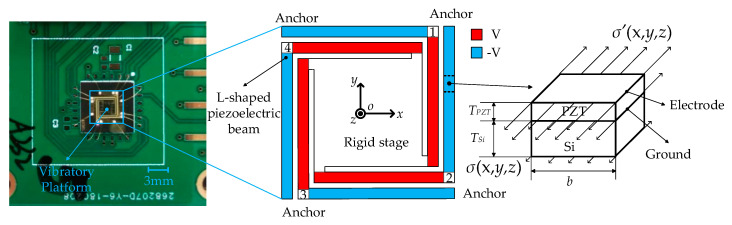
Piezoelectric vibratory platform.

The vibratory platform is shown in Figure 2. It consists of a rigid stage supported by four L-shape beams which were constructed using a silicon structure layer, a lead zirconate titanate (PZT) drive layer, and two metal electrode layers. The movement along the *z*-axis was actuated by a designed combination of positive voltage on the inner parts and negative voltage on the outer parts.

**Figure 3:**
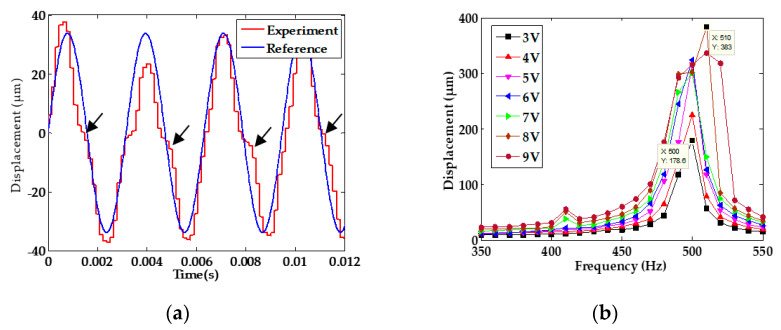
(**a**) Displacement response with abnormal phenomenon; (**b**) amplitude-frequency characteristic.

The excitation voltage was a sinusoidal signal with an amplitude of 8 V and frequency of 317 Hz. The test results indicate an obvious offset near the zero point of displacement in Figure 3a, and when different voltages with amplitudes from 3 V to 9 V were applied to acquire the amplitude-frequency curves, an increase of resonant frequency of stage from 500 Hz to 510 Hz appeared, as shown in Figure 3b. The abnormal phenomenon could be induced from the residual stress [22] in each L-shape beam, as shown the right side of Figure 1, PZT hysteresis, or both [23]. The following will describe our investigation of the influence of both mechanisms.

## 3. Investigation of the Abnormal Phenomenon

### 3.1. Influence of Residual Stress

**Figure 4:**
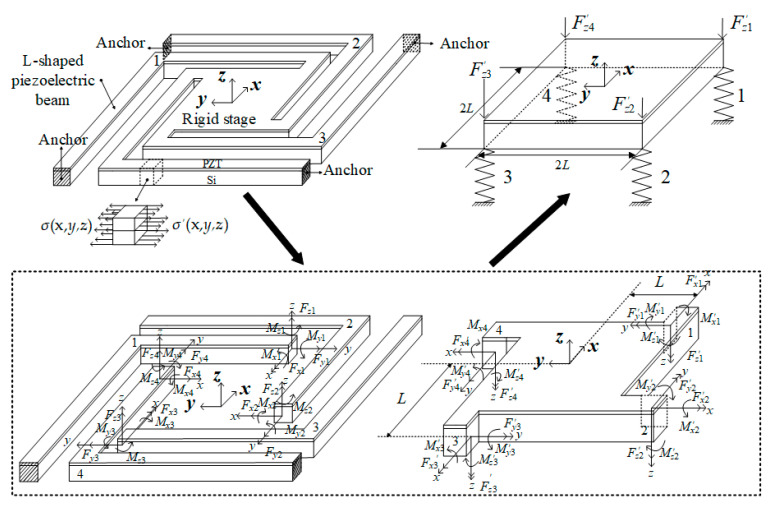
The relationship between rigid stage and four L-shaped beams.

The residual stress of the beam for supporting the vibratory platform is caused by the micro fabrication processes, like deposition, etching, sputtering, releasing, and so on. It induces an initial displacement of the vibratory platform. Before that, the four L-shaped beams of the MEMS micro vibratory platform are connected with each other to form a closed ring beam. The relationship between each part can be seen in Figure 4.

The vibratory platform can be parted with four L-shaped beams and a rigid stage. The in-plane deformation of the beam is negligible when compared to the out-of-plane deformation. Due to the symmetry of structure and load, only one single L-shape beam needs to be modeled. One end of beam fixed on the substrate had the boundary condition of all the displacement and rotation equal to zero, and the other end, connecting to the stage, has a zero rotation and a single translation freedom along *z*-axis, called a guided boundary condition [24,25].

**Figure 5:**
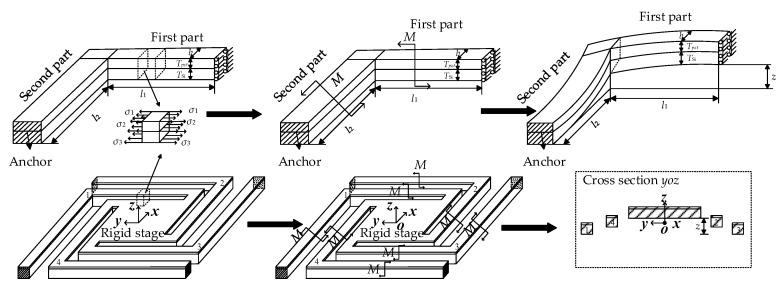
Deformation diagram of L-shaped piezoelectric micro beam and vibratory platform with inside residual stress.

Because of the elastic deformation of materials, the stress gradient is linear within each layer of L-shaped beam [26]. The stress in the top and bottom layer of the beam is assumed to be *σ*_1_ and *σ*_3_, and the stress at the interface between the piezoelectric layer and the silicon layer is *σ*_2_ (Figure 5).

The total stress of beam expresses can be expressed as:

(1) $$stress=\frac{\sigma_{1}+\sigma_{2}}{2}\frac{T_{PZT}}{T_{PZT}+T_{si}}+\frac{\sigma_{2}+\sigma_{3}}{2}\frac{T_{si}}{T_{PZT}+T_{si}}+\left(\sigma_{1}-\sigma_{2}\right)\frac{z-T_{si}}{T_{PZT}}|{}_{T_{si}\leq z\leq T_{pzt}+T_{si}}+\left(\sigma_{2}-\sigma_{3}\right)\frac{z}{T_{Si}}|{}_{0\leq z<T_{si}},$$

where the *T_PZT_* and *T_Si_* represent the thickness of PZT layer and Si layer, respectively. The bottom of the beam as the zero point of the z coordinate. The uniform stress (the first two items in Equation (1)), *T_pzt_* (σ_1+_σ_2_)/ (2(*T_pzt_*+*T_Si_*))+ *T_Si_* (σ_2+_σ_3_)/(2(*T_pzt_*+*T_Si_*)) accounts for the in-plane elongation of the beam, whereas the (σ_1_-σ_2_)((*z*-*T_Si_*)/*T_pzt_*) + (σ_2_-σ_3_)(*z*/*T_Si_*) (the stress gradient component, the last two items in Equation (1)) causes out-of-plane deflection. The equivalent bending moment due to residual stress can be written as:

(2) $$\begin{array}{l} M=\int_{0}^{T_{PZT}+T_{Si}}dM\\ \;=\int_{T_{si}}^{T_{PZT}+T_{Si}}\left(\sigma_{1}-\sigma_{2}\right)\frac{b{\left(z-T_{si}\right)}^{2}}{T_{PZT}}dz+\int_{0}^{Tsi}\left(\sigma_{2}-\sigma_{3}\right)\frac{bz^{2}}{T_{Si}}dz\\ \;=\frac{b\left(\sigma_{1}-\sigma_{2}\right)}{T_{PZT}}\left(\frac{{\left(T_{PZT}+T_{Si}\right)}^{3}}{3}-T_{PZT}{}^{2}T_{Si}-T_{PZT}T_{Si}{}^{2}\right)+\frac{b\left(\sigma_{2}-\sigma_{3}\right)}{T_{Si}}\frac{T_{Si}^{3}}{3}, \end{array}$$

where *b* is the width of beam. The equivalent bending moment, *M*, is a linear combination of stress gradient due to residual stress. Then, a beam theory was used to calculate the beam initial deflection [27] by:

(3) $$EI\frac{d^{2}z\left(y\right)}{dy^{2}}=M,$$

(4) $$z\left(y\right)=\left[\frac{b\left(\sigma_{1}-\sigma_{2}\right)}{T_{PZT}}\left(\frac{{\left(T_{PZT}+T_{Si}\right)}^{3}}{3}-T_{PZT}{}^{2}T_{Si}-T_{PZT}T_{Si}{}^{2}\right)+\frac{b\left(\sigma_{2}-\sigma_{3}\right)}{T_{Si}}\frac{T_{Si}^{3}}{3}\right]\frac{y^{2}}{EI}+C_{1}y+C_{2},$$

where the *C*_1_ and *C*_2_ are decided by specific boundary conditions. Substituting *y* = *l_i_*, (*l*_i_ is the length of *i*-th part of each L-shaped beam) into Equation (4), the initial deflection of the beam can be expressed as:

(5) $$\begin{array}{ll} z_{initial}\left(l_{i}\right) & =\left(\sigma_{1}-\sigma_{2}\right)\left[\frac{bl^{2}}{T_{PZT}EI}\left(\frac{{\left(T_{PZT}+T_{Si}\right)}^{3}}{3}-T_{PZT}{}^{2}T_{Si}-T_{PZT}T_{Si}{}^{2}\right)\right]\\ & +\left(\sigma_{2}-\sigma_{3}\right)\left[\frac{bl^{2}}{T_{PZT}EI}\frac{T_{Si}^{3}}{3}\right]\\ & +C_{1}l_{i}+C_{2}. \end{array}$$

**Figure 6:**
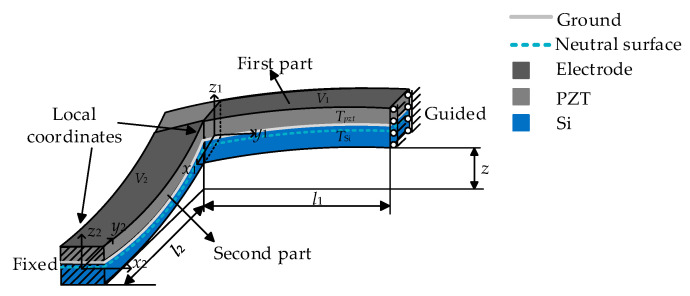
L-shaped micro beam diagram with the initial deflection *z*.

The initial deflection *z_initial_* (*l_i_*) of the piezoelectric micro beam is a linear combination of residual stress gradient and *z* = *z_initial_* (*l*_1_) +*z_initial_* (*l*_2_). The obvious difference of curvatures of two segments results from the distinguished load and end constraints. Thus, the residual stress is replaced by the initial deflection *z* to investigate the impacts of displacement response (Figure 6).

**Figure 7:**
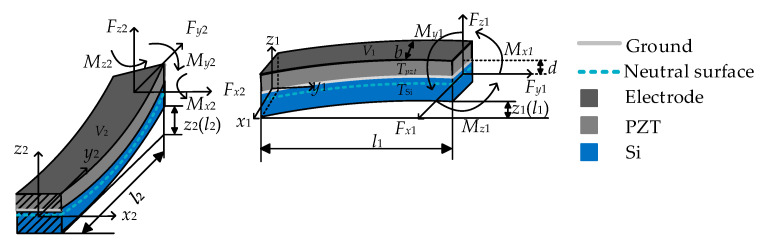
Diagram of each segment beam.

The L-shaped beam is divided into two segments for a simple analysis. When one segment is considered, the other is treated as a rigid body. The guided boundary can be replaced by a set of *F_x_*, *F_y_*, *F_z_*, *M_x_*, *M_y_*, and *M*, and the *F_xi_*, *F_yi_*, *F_zi_*, *M_xi_*, *M_yi_*, and *M_zi_* represent the set of force and moment at the end of each part of the L-shaped beam respectively, where the subscript *i* indicates the *i*-th part of L-shaped beam (Figure 7).

The relationship between *F_x_*, *F_y_*, *F_z_*, *M_x_*, *M_y_*, *M_z_* and *F_xi_*, *F_yi_*, *F_zi_*, *M_xi_*, *M_yi_*, and *M_zi_* are shown in Equation (6):

(6) $$\begin{array}{l} F_{x1}=F_{x},\;F_{y1}=F_{y},\;F_{z1}=F_{z},\;M_{x1}=M_{x},\;M_{y1}=M_{y},\;M_{z1}=M_{z}\\ F_{x2}=F_{y},\;F_{y2}=-F_{x},\;F_{z2}=F_{z},\;M_{x2}=M_{y},\;M_{y2}=-M_{x}+F_{z}l_{1},\;M_{z2}=M_{z}-F_{x}l_{1} \end{array}$$

The stiffness, *F_z_*/*z*, is regarded as the solution by the following constraints at the corner of the L-shaped beam in Equation (7).

(7) $$x_{1}(0)=y_{2}(l_{2}),\;y_{1}(0)=x_{2}(l_{2}),\;z_{1}(0)=z_{2}(l_{2}),\;\theta_{x1}(0)=-\theta_{y2}(l_{2}),\;\theta_{y1}(0)=\theta_{x2}(l_{2}),\;\theta_{z1}(0)=\theta_{z2}(l_{2}),$$

where *x_i_*, *y_i_* and *z_i_* represent the displacement of the *i*-th part on the *x*-axis, *y*-axis, and *z*-axis, respectively. *θ_xi_*, *θ_yi_* and *θ_zi_* represent the angle of the *i*-th part around the *x*-axis, *y*-axis, and *z*-axis, respectively. Then, the differential equation of deflection is given by [28] as:

(8) $$EI_{i}\frac{d^{2}z_{i}\left(y_{i}\right)}{dy_{i}^{2}}=F_{zi}\left(l_{i}-y_{i}\right)+M_{xi}-dE_{pzt}bd_{31}V_{i}-F_{yi}\left[z_{i}(l_{i})-z_{i}(y_{i})\right],$$

In Equation (8), the *E_pzt_* is the elastic modulus of PZT. *EI_i_* is the flexural rigidity. *d* is the distance between the PZT layer center coordinate and the neutral surface. *b* is the width of beam, and *d*_31_ is the piezoelectric coefficient [29]. *V_i_* is the driving voltage.

The solution of the differential equation is shown in Equations (9) and (10):

(9) $$z_{i}\left(y_{i}\right)|{}_{\alpha_{i1}<0}=C_{i1}\frac{F_{zi}}{EI_{i}}\cos\left(\sqrt{-\alpha_{i1}}y_{i}\right)+C_{i2}\frac{F_{zi}}{EI_{i}}\sin\left(\sqrt{-\alpha_{i1}}y_{i}\right)+\frac{\frac{F_{zi}}{EI_{i}}y_{i}-\alpha_{i1}z_{i}\left(l_{i}\right)-\frac{F_{zi}}{EI_{i}}l_{i}-C_{i3}}{-\alpha_{i1}},$$

(10) $$z_{i}\left(y_{i}\right)|{}_{\alpha_{i1}>0}=C_{i1}\frac{F_{zi}}{EI_{i}}e^{\sqrt{\alpha_{i1}}y_{i}}+C_{i2}\frac{F_{zi}}{EI_{i}}e^{-\sqrt{\alpha_{i1}}y_{i}}+\frac{\frac{F_{zi}}{EI_{i}}y_{i}+\alpha_{i1}z_{i}\left(l_{i}\right)-\frac{F_{zi}}{EI_{i}}l_{i}-C_{i3}}{\alpha_{i1}},$$

where α*_i_*_1_ = *F_yi_*/*EI_i_*, the unknown coefficients C*_i_*_1_, C*_i_*_2_, and C *_i_*_3_ were listed in Table 1, parameter *k_ri_* is the torsional stiffness of the *i*-th part.

For brevity, the unknown coefficient C *_i_*_3_ can be abbreviated as C *_i_*_3_ = *A_i_*_3_ *F_zi_/EI_i_* + *B_i_*_3_ *z_i_* (*l_i_*). Substituting *y_i_* = *l*_i_ into Equations (9) and (10), the result can be expressed in Equation (11).

(11) $$\left\{ \begin{array}{l} \frac{F_{zi}}{z_{i}}|{}_{F_{yi}>0}=B_{i3}{\left[\alpha_{i1}\left(\frac{C_{i1}}{EI_{i}}e^{\sqrt{\alpha_{i1}}l_{i}}+\frac{C_{i2}}{EI_{i}}e^{-\sqrt{\alpha_{i1}}l_{i}}-\frac{A_{i3}}{EI_{i}\alpha_{i1}}\right)\right]}^{-1}\\ \frac{F_{zi}}{z_{i}}|{}_{F_{yi}<0}=B_{i3}{\left[\alpha_{i1}\left(\frac{C_{i1}}{EI_{i}}\cos\left(\sqrt{-\alpha_{i1}}l_{i}\right)+\frac{C_{i2}}{EI_{i}}\sin\left(\sqrt{-\alpha_{i1}}l_{i}\right)-\frac{A_{i3}}{EI_{i}\alpha_{i1}}\right)\right]}^{-1} \end{array}\right.$$

**Figure 8:**
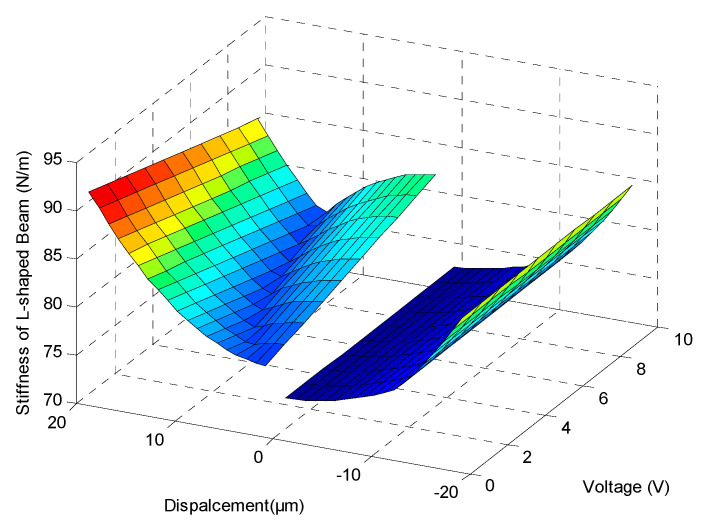
Stiffness of L-shaped piezoelectric beam.

It can be seen that Equation (11) is a piecewise function (*α_i_*_1_ = *F_yi_*/*EI_i_*); it is related to the axial force *F_yi_* which is determined by the beam initial deflection *z*, vibration displacement, and driving voltage. The relationship between stiffness and displacement under different voltages is depicted in Figure 8.

There was a sharp increase of stiffness around zero displacement, because the axial force, *F_yi_*, changed from a compressive state of negative displacement to a tensile state of positive displacement due to the residual stress. The level of stiffness variation is also related to the driving voltage as the internal stress level results from the piezoelectric layer deformation. The stiffening effect also appeared in the stage of large deformation of the beam and lead to a higher frequency of the vibratory platform.

### 3.2. Modeling PZT Hysteresis

**Figure 9:**
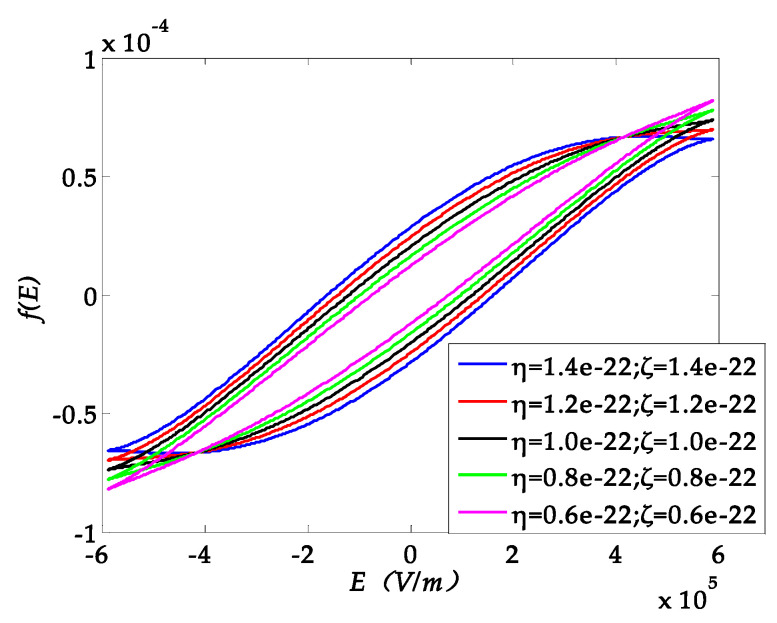
Hysteresis loop function with different parameters *η* and *ζ*.

The PZT hysteresis is another factor possibly resulting in a displacement error. The hysteretic behavior of piezoelectric material under the voltage stimulus is reported in [30,31]. To model this mechanism, the polynomial-based hysteresis model is simple and suitable to analyze the dynamic response of the system [32,33], which can be expressed as Equation (12):

(12) $$f\left(E\right)=d_{ij}E-\eta E^{3}+\frac{\zeta }{\omega^{3}}{\dot{E}}^{3},$$

where the *f (E)* is the hysteresis loop function. *d_ij_* is the piezoelectric coefficient. *E* is the electric field, and *ω* is the frequency. The parameters *η* and *ζ* are the fitting parameters determining the shape of the hysteresis loop (Figure 9).

In this example, the electric field is *E* = *V_max_* sin (*ωt*)/*T_PZT_*, where *V_max_* is the maximum amplitude of voltage, 10 V, and *T_PZT_* is the thickness of the piezoelectric layer, 17μm. The hysteresis loop function f(E) is described as the scaling behavior [34] of hysteresis in PZT ceramic by different η and ζ. 

### 3.3. Residual Stress vs. PZT Hysteresis

**Figure 10:**
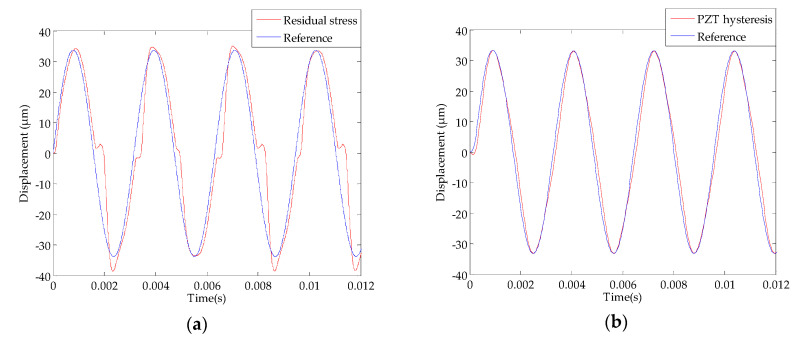
Displacement response simulation 8 V (317 Hz); (**a**) residual stress; (**b**) lead zirconate titanate (PZT) hysteresis.

Based on the mechanisms described above and the electromechanical coupling model reported in previous work, the commercial software Simulink was utilized to simulate the displacement response of vibratory platform. While the vibratory platform can be regarded as a mass-spring-damping system, and its vibration equation is shown in Equation (13).

(13) $$M\ddot{z}+C\dot{z}+Kz=K_{f}\left(d_{ij}E-\eta E^{3}+\frac{\zeta }{\omega^{3}}{\dot{E}}^{3}\right),$$

where *M* and *C* represent the equivalent mass and damping coefficient separately, *K* is the stiffness in Equation (12). The right side of the equation represents the equivalent driving force which contains the hysteresis loop function, and the *K_f_* is the coefficient. In Figure 10a,b show the influence of the residual stress and the PZT hysteresis, respectively. It indicates that the offset of the zero point is attributed to the residual stress and has almost nothing to do with the hysteresis of PZT materials (Figure 10b).

**Figure 11:**
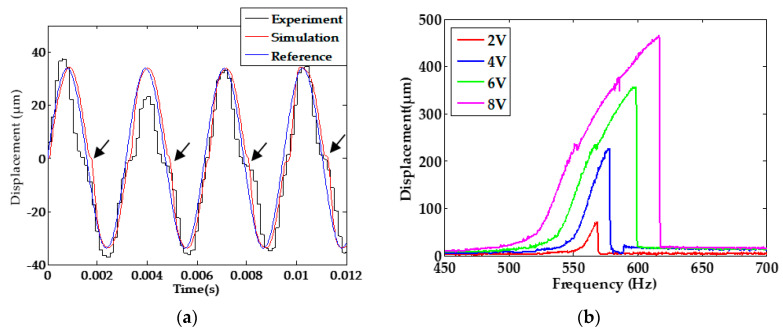
(**a**) Displacement response at sinusoidal excitation 8 V (317 Hz); (**b**) Amplitude-frequency response.

For a comparison, Figure 11 considered both the residual stress and PZT hysteresis to investigate the impacts on the platform. The experiments and theoretical/simulation show agreement with each other.

## 4. Discussion

**Figure 12:**
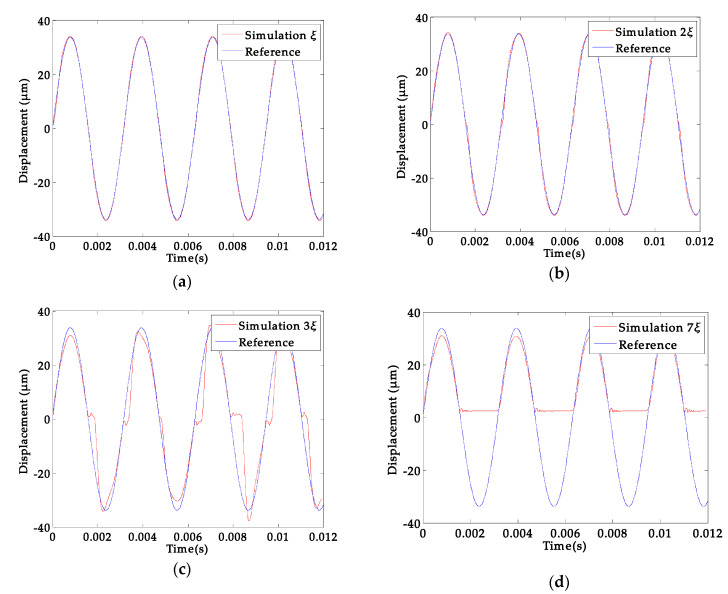
Displacement response simulation under different residual stress gradient 8 V (317 Hz); (**a**) *ξ* stress gradient; (**b**) 2*ξ* stress gradient; (**c**) 3*ξ* stress gradient; (**d**) 7*ξ* stress gradient.

In Section 3, the different stress gradient and load condition are discussed for the abnormal phenomena of the platform vibration. We set the linear stress gradient to *ξ*, and then the results of different stress gradients were simulated, as can be seen in Figure 12.

The increase of the stress gradient caused the apparent abnormal phenomenon that can be seen in Figure 12. When the residual stress gradient increased to a certain level, the platform even failed (Figure 12d).

**Figure 13:**
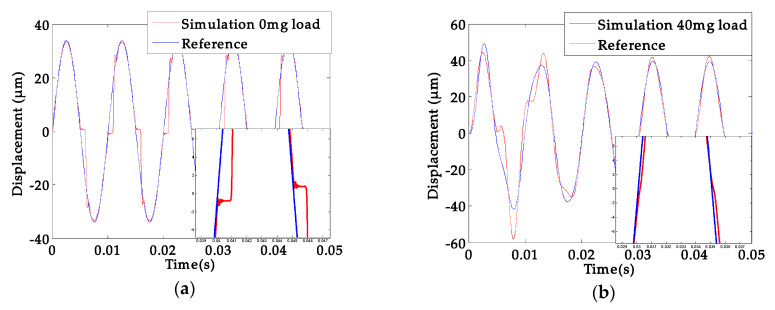

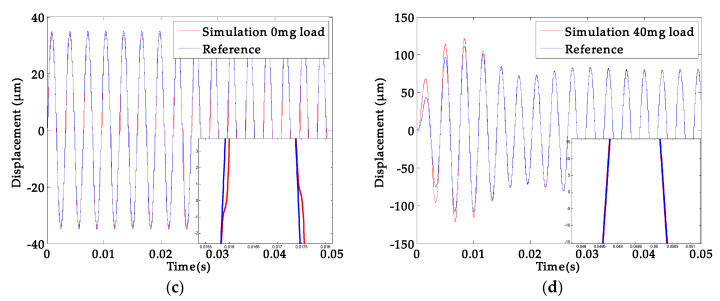
Displacement response simulation; (**a**) 0 mg load under 8 V (100 Hz); (**b**) 40 mg load under 8 V (100 Hz); (**c**) 0 mg load under 8 V (317 Hz); (**d**) 0 mg load under 8 V (317 Hz).

Moreover, whether the platform was loaded or not also affected the displacement response. The cases of unload or with a 40 mg load can be seen in Figure 13.

In Figure 13, when the 40 mg load is fixed on the platform, its first-order natural frequency decreased, which made the vibration state of platform closer to the resonance state. Although the loaded platform (40 mg) weakened its abnormal displacement response, this abnormal phenomenon was still obvious under the low frequency working state, which seriously affected the calibration accuracy across the full frequency range.

**Figure 14:**
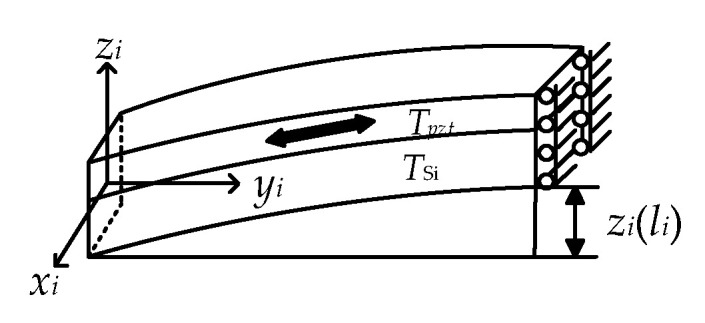
Each part diagram of L-shaped micro beam with the initial deflection *z_i_* (*l_i_*).

The displacement deviation induced by residual stress could be explained as follows. The fabricated beam exhibited an initial deflection *z_i_* (*l_i_*) (Figure 14). When the stage moved to the zero position, more strain energy was needed to overcome the resistance of compressing beam and the axial load of PZT layer under the driving voltage, and therefore the needed energy from actuation costs more time than normal state, which shows an abnormal of movement through zero position.

## 5. Conclusions

In this study, the observed displacement response errors were investigated theoretically. It was concluded that both the displacement offset and increased frequency are caused by the residual stress from microfabrication, rather than the hysteresis of PZT materials. The initial deflection induced by residual stress makes the stage need more electrical energy to conquer the resistance of axial strain. Therefore, a good recalibration accuracy can be obtained by stress-free supporting beams or using the stimulus far from the zero point to calibrate the inertial sensors.

## Figures and Tables

**Table 1:** The coefficients of Equations (9) and (10).

Coefficient	Expression
*α*_11_ < 0, *C*_11_	$\frac{\left(\cos\left(\sqrt{-\alpha_{11}}l_{1}\right)-1\right)}{-\alpha_{11}^{3/2}\left(\sin\left(\sqrt{-\alpha_{11}}l_{1}\right)+\frac{EI_{1}}{kr_{1}}\sqrt{-\alpha_{11}}\cos\left(\sqrt{-\alpha_{11}}l_{1}\right)\right)}$
*α*_11_ < 0, *C*_12_	$\frac{\left(\sin\left(\sqrt{-\alpha_{11}}l_{1}\right)+\frac{EI_{1}}{kr_{1}}\sqrt{-\alpha_{11}}\right)}{-\alpha_{11}^{3/2}\left(\sin\left(\sqrt{-\alpha_{11}}l_{1}\right)+\frac{EI_{1}}{kr_{1}}\sqrt{-\alpha_{11}}\cos\left(\sqrt{-\alpha_{11}}l_{1}\right)\right)}$
*α*_11_ < 0, *C*_13_	$\begin{array}{l} \frac{F_{z1}}{EI_{1}}\frac{\left(1-\cos\left(\sqrt{-\alpha_{11}}l_{1}\right)\right)-\sqrt{-\alpha_{11}}l_{1}\sin\left(\sqrt{-\alpha_{11}}l_{1}\right)-\left(-\alpha_{11}\right)l_{1}\frac{EI_{1}}{kr_{1}}\cos\left(\sqrt{-\alpha_{11}}l_{1}\right)}{-\alpha_{11}^{1/2}\left(\sin\left(\sqrt{-\alpha_{11}}l_{1}\right)+\frac{EI_{1}}{kr_{1}}\sqrt{-\alpha_{11}}\cos\left(\sqrt{-\alpha_{11}}l_{1}\right)\right)}\\ +z_{1}\left(l_{1}\right)\frac{-\alpha_{11}^{3/2}\sin\left(\sqrt{-\alpha_{11}}l_{1}\right)+\alpha_{11}^{2}\frac{EI_{1}}{kr_{1}}\cos\left(\sqrt{-\alpha_{11}}l_{1}\right)}{-\alpha_{11}^{1/2}\left(\sin\left(\sqrt{-\alpha_{11}}l_{1}\right)+\frac{EI_{1}}{kr_{1}}\sqrt{-\alpha_{11}}\cos\left(\sqrt{-\alpha_{11}}l_{1}\right)\right)} \end{array}$
*α*_21_ < 0, *C*_21_	$-\frac{\left(1-\cos\left(\sqrt{-\alpha_{21}}l_{2}\right)\right)+\sqrt{-\alpha_{21}}\frac{EI_{2}}{kr_{2}}\sin\left(\sqrt{-\alpha_{21}}l_{2}\right)}{-\alpha_{21}^{3/2}\left(\sin\left(\sqrt{-\alpha_{21}}l_{2}\right)+\frac{EI_{2}}{kr_{2}}\sqrt{-\alpha_{21}}\cos\left(\sqrt{-\alpha_{21}}l_{2}\right)\right)}$
*α*_21_ < 0, *C*_22_	$\frac{1}{-\alpha_{21}^{3/2}}$
*α*_21_ < 0, *C*_23_	$\begin{array}{l} \frac{F_{z2}}{EI_{2}}\left[\begin{array}{l} \frac{\left(1-\cos\left(\sqrt{-\alpha_{21}}l_{2}\right)\right)+\sqrt{-\alpha_{21}}\frac{EI_{2}}{kr_{2}}\sin\left(\sqrt{-\alpha_{21}}l_{2}\right)}{-\alpha_{21}^{1/2}\left(\sin\left(\sqrt{-\alpha_{21}}l_{2}\right)+\frac{EI_{2}}{kr_{2}}\sqrt{-\alpha_{21}}\cos\left(\sqrt{-\alpha_{21}}l_{2}\right)\right)}\\ -\frac{\sqrt{-\alpha_{21}}l_{2}\sin\left(\sqrt{-\alpha_{21}}l_{2}\right)+\left(-\alpha_{21}\right)l_{2}\frac{EI_{2}}{kr_{2}}\cos\left(\sqrt{-\alpha_{21}}l_{2}\right)}{-\alpha_{21}^{1/2}\left(\sin\left(\sqrt{-\alpha_{21}}l_{2}\right)+\frac{EI_{2}}{kr_{2}}\sqrt{-\alpha_{21}}\cos\left(\sqrt{-\alpha_{21}}l_{2}\right)\right)} \end{array}\right]\\ +z_{2}\left(l_{2}\right)\frac{-\alpha_{21}^{3/2}\sin\left(\sqrt{-\alpha_{21}}l_{2}\right)+\alpha_{21}^{2}\frac{EI_{2}}{kr_{2}}\cos\left(\sqrt{-\alpha_{21}}l_{2}\right)}{-\alpha_{21}^{1/2}\left(\sin\left(\sqrt{-\alpha_{21}}l_{2}\right)+\frac{EI_{2}}{kr_{2}}\sqrt{-\alpha_{21}}\cos\left(\sqrt{-\alpha_{21}}l_{2}\right)\right)} \end{array}$
*α*_11_ > 0, *C*_11_	$-\frac{e^{\sqrt{\alpha_{11}}l_{1}}-1+\sqrt{\alpha_{11}}\frac{EI_{1}}{kr_{1}}e^{\sqrt{\alpha_{11}}l_{1}}}{\alpha_{11}^{3/2}\left(e^{2\sqrt{\alpha_{11}}l_{1}}+\sqrt{\alpha_{11}}\frac{EI_{1}}{kr_{1}}+\sqrt{\alpha_{11}}\frac{EI_{1}}{kr_{1}}e^{2\sqrt{\alpha_{11}}l_{1}}-1\right)}$
*α*_11_ > 0, *C*_12_	$\frac{e^{\sqrt{\alpha_{11}}l_{1}}\left(e^{\sqrt{\alpha_{11}}l_{1}}+\sqrt{\alpha_{11}}\frac{EI_{1}}{kr_{1}}-1\right)}{\alpha_{11}^{3/2}\left(e^{2\sqrt{\alpha_{11}}l_{1}}+\sqrt{\alpha_{11}}\frac{EI_{1}}{kr_{1}}+\sqrt{\alpha_{11}}\frac{EI_{1}}{kr_{1}}e^{2\sqrt{\alpha_{11}}l_{1}}-1\right)}$
*α*_11_ > 0, *C*_13_	$\begin{array}{l} \frac{F_{z1}}{EI_{1}}\frac{1+\sqrt{\alpha_{11}}l_{1}-2e^{\sqrt{\alpha_{11}}l_{1}}+e^{2\sqrt{\alpha_{11}}l_{1}}-\sqrt{\alpha_{11}}l_{1}e^{2\sqrt{\alpha_{11}}l_{1}}-\alpha_{11}l_{1}\frac{EI_{1}}{kr_{1}}-\alpha_{11}l_{1}\frac{EI_{1}}{kr_{1}}e^{2\sqrt{\alpha_{11}}l_{1}}}{\sqrt{\alpha_{11}}\left(e^{2\sqrt{\alpha_{11}}l_{1}}+\sqrt{\alpha_{11}}\frac{EI_{1}}{kr_{1}}+\sqrt{\alpha_{11}}\frac{EI_{1}}{kr_{1}}e^{2\sqrt{\alpha_{11}}l_{1}}-1\right)}\\ +z_{1}\left(l_{1}\right)\frac{\alpha_{11}^{3/2}\left(1-e^{2\sqrt{\alpha_{11}}l_{1}}\right)+\alpha_{11}^{2}\frac{EI_{1}}{kr_{1}}+\alpha_{11}^{2}\frac{EI_{1}}{kr_{1}}e^{2\sqrt{\alpha_{11}}l_{1}}}{\sqrt{\alpha_{11}}\left(e^{2\sqrt{\alpha_{11}}l_{1}}+\sqrt{\alpha_{11}}\frac{EI_{1}}{kr_{1}}+\sqrt{\alpha_{11}}\frac{EI_{1}}{kr_{1}}e^{2\sqrt{\alpha_{11}}l_{1}}-1\right)} \end{array}$
*α*_21_ > 0, *C*_21_	$-\frac{e^{\sqrt{\alpha_{21}}l_{2}}-1+\sqrt{\alpha_{21}}\frac{EI_{2}}{kr_{2}}}{\alpha_{21}^{3/2}\left(e^{2\sqrt{\alpha_{21}}l_{2}}+\sqrt{\alpha_{21}}\frac{EI_{2}}{kr_{2}}+\sqrt{\alpha_{21}}\frac{EI_{2}}{kr_{2}}e^{2\sqrt{\alpha_{21}}l_{2}}-1\right)}$
*α*_21_ > 0, *C*_2__2_	$\frac{e^{2\sqrt{\alpha_{21}}l_{2}}-e^{\sqrt{\alpha_{21}}l_{2}}+\sqrt{\alpha_{21}}\frac{EI_{2}}{kr_{2}}e^{2\sqrt{\alpha_{21}}l_{2}}}{\alpha_{21}^{3/2}\left(e^{2\sqrt{\alpha_{21}}l_{2}}+\sqrt{\alpha_{21}}\frac{EI_{2}}{kr_{2}}+\sqrt{\alpha_{21}}\frac{EI_{2}}{kr_{2}}e^{2\sqrt{\alpha_{21}}l_{2}}-1\right)}$
*α*_21_ > 0, *C*_23_	$\begin{array}{l} \frac{F_{z2}}{EI_{2}}\frac{{\left(e^{\sqrt{\alpha_{21}}l_{2}}-1\right)}^{2}+\sqrt{\alpha_{21}}\frac{EI_{2}}{kr_{2}}\left(e^{2\sqrt{\alpha_{21}}l_{2}}-1\right)-\sqrt{\alpha_{21}}l_{2}\left(e^{2\sqrt{\alpha_{21}}l_{2}}-1\right)+\alpha_{21}l_{2}\frac{EI_{2}}{kr_{2}}\left(e^{2\sqrt{\alpha_{21}}l_{2}}+1\right)}{\sqrt{\alpha_{21}}\left(e^{2\sqrt{\alpha_{21}}l_{2}}+\sqrt{\alpha_{21}}\frac{EI_{2}}{kr_{2}}+\sqrt{\alpha_{21}}\frac{EI_{2}}{kr_{2}}e^{2\sqrt{\alpha_{21}}l_{2}}-1\right)}\\ +z_{2}\left(l_{2}\right)\frac{\alpha_{21}^{3/2}\left(e^{2\sqrt{\alpha_{21}}l_{2}}-1\right)+\alpha_{21}^{2}\frac{EI_{2}}{kr_{2}}\left(e^{2\sqrt{\alpha_{21}}l_{2}}+1\right)}{\sqrt{\alpha_{21}}\left(e^{2\sqrt{\alpha_{21}}l_{2}}+\sqrt{\alpha_{21}}\frac{EI_{2}}{kr_{2}}+\sqrt{\alpha_{21}}\frac{EI_{2}}{kr_{2}}e^{2\sqrt{\alpha_{21}}l_{2}}-1\right)} \end{array}$
